# Anticancer Potential of Selected Flavonols: Fisetin, Kaempferol, and Quercetin on Head and Neck Cancers

**DOI:** 10.3390/nu13030845

**Published:** 2021-03-05

**Authors:** Robert Kubina, Marcello Iriti, Agata Kabała-Dzik

**Affiliations:** 1 Department of Pathology, Faculty of Pharmaceutical Sciences in Sosnowiec, Medical University of Silesia, 40-055 Katowice, Poland; adzik@sum.edu.pl; 2 Department of Agricultural and Environmental Sciences, Milan State University, via G. Celoria 2, 20133 Milan, Italy; marcello.iriti@unimi.it

**Keywords:** flavonols, head and neck cancer prevention, molecular mechanism

## Abstract

Flavonols are ones of the most common phytochemicals found in diets rich in fruit and vegetables. Research suggests that molecular functions of flavonoids may bring a number of health benefits to people, including the following: decrease inflammation, change disease activity, and alleviate resistance to antibiotics as well as chemotherapeutics. Their antiproliferative, antioxidant, anti-inflammatory, and antineoplastic activity has been proved. They may act as antioxidants, while preventing DNA damage by scavenging reactive oxygen radicals, reinforcing DNA repair, disrupting chemical damages by induction of phase II enzymes, and modifying signal transduction pathways. One of such research areas is a potential effect of flavonoids on the risk of developing cancer. The aim of our paper is to present a systematic review of antineoplastic activity of flavonols in general. Special attention was paid to selected flavonols: fisetin, kaempferol, and quercetin in preclinical and in vitro studies. Study results prove antiproliferative and proapoptotic properties of flavonols with regard to head and neck cancer. However, few study papers evaluate specific activities during various processes associated with cancer progression. Moreover, an attempt was made to collect the majority of substantive studies on bioactive potential of the selected flavonols, especially with regard to modulation of a range of signal transduction pathways that participate in cancer development.

## 1. Introduction

Morbidity of neoplastic diseases and cancer mortality increase at an accelerated pace in both developed and developing countries. New Globocan 2020 data regarding cancer and including 185 countries indicated over 19 million new cancer cases and over 9.8 million deaths associated with cancer. In spite of medical advancements in diagnostics and treatment of neoplastic diseases, cancers remain the most common cause of death around the world [1]. Limited funding for prophylactic measures (including prophylaxis against neoplastic diseases) due to low budget is both a Europe-wide and worldwide problem. Successful strategies for dealing with threats such as cancer are needed. Flavonoids are well-known plant products and, according to numerous reports, possess biological activities directed against many diseases, such as malignant neoplasms, including head and neck cancer.

Head and neck squamous cell carcinomas (HNSCC) include, first and foremost, cancers that develop in the upper part of the respiratory tract and the gastrointestinal tract. Global statistics indicate that HNSCC occur worldwide with the frequency of 500,000 cases per year. In the recent years, a considerable increase in morbidity and mortality caused by malignant neoplasms, including head and neck cancer, has been observed. All epithelial tumors constitute a great challenge in clinical practice; however, it is the anatomy of tumors in the area of head and neck that makes them especially difficult to treat. Even though novel methods have improved locoregional control in patients with advanced HNSCC, locoregional, and distant recurrences are common and almost always lead to death. Hence, it is of the essence to improve systemic therapies of patients with such tumors in order to increase the cure rate and decrease the morbidity rate [2].

## 2. Flavonols

Structure of Flavonols

**Figure 1:**
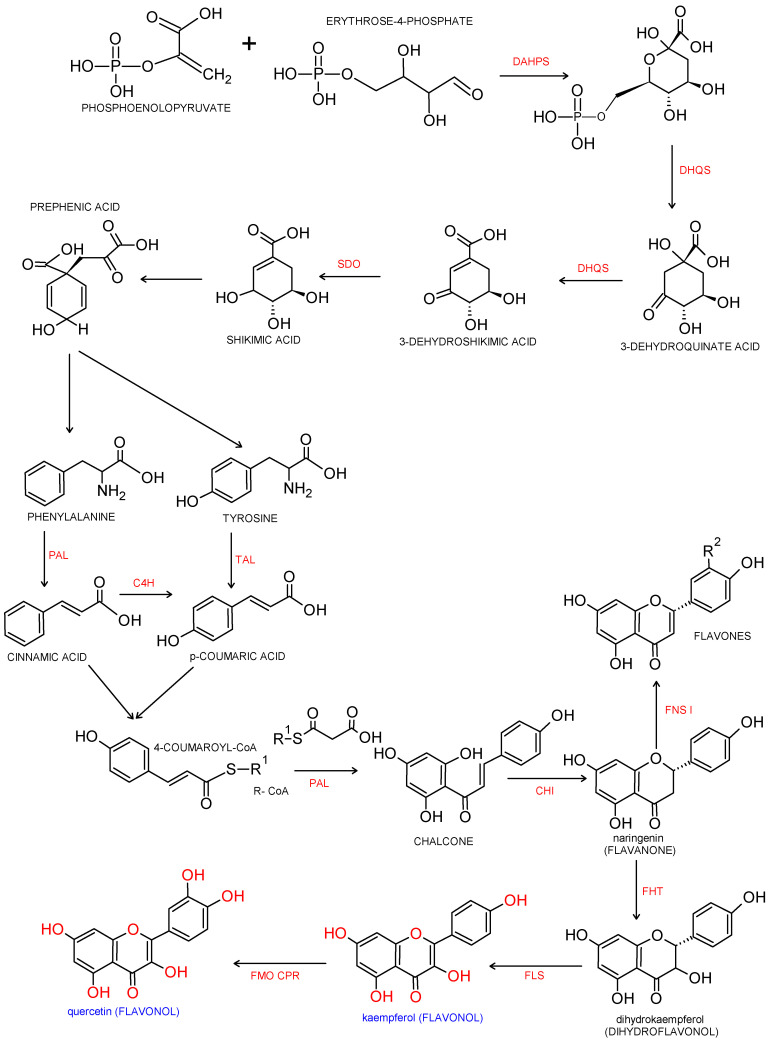
Biosynthesis pathway of phenylpropanoids and flavonols. Biosynthesis of flavonols from p-coumaroyl-CoA and malonyl-CoA. Enzymatic reaction catalyzed by DAHPS: 3-deoxy-D-arabino-heptulosonate-7-phosphate-synthase; DHQS: 3-dexydroquinate synthase; SDO: shikimate dehydrogenase; PAL: phenylalanine ammonia-lyase; TAL: tyrosine ammonia lyase; C4H: cinnamate 4-hydroxylase; CHI: chalcone isomerase, FHT: flavanone 3β-hydroxylase; FNS Ι: flavone synthase Ι; FLS: flavonol synthase; FMO: flavonoid 3′-monooxygenase; CPR: cytochrome P450reductase [8,9,10].

Flavonols are a major class of the family of flavonoids. Flavonoids are 2 phenyl benzo-γ-pyrone derivatives. A common part in the chemical structure of all flavonoids is a 15-carbon skeleton based on a flavan structure (C6-C3-C6) consisting of two aromatic rings (A and B) linked by a heterocyclic pyrane or pyrone ring (C). A wide diversity of flavonoids stems from the fact that carbon atoms of A, B and C rings may undergo hydroxylation, methoxylation and glycosidation by means of mono- and oligosaccharides, as well as acylation at different positions. Biogenesis of flavonoids is a complex process. They are created from precursors of primary metabolism during a transformation creating two pathways, as shown in Figure 1. The first one is a malonic acid pathway, in which malonyl coenzyme A is created from acetyl coenzyme A produced through glycolysis; three molecules of malonyl-CoA obtained from glucose transformation are the source of the aromatic ring A. The second one is a shikimic acid pathway, in which erythrose-4-phosphate derived from the pentose phosphate pathway and phosphoenolpyruvate created via glycolysis are transformed into shikimic acid, and then, into aromatic amino acids: phenylalanine and tyrosine. During subsequent transformations, both amino acids undergo deamination, and consequently, form cinnamic acid. Condensation of ring A and ring B results in creation of chalcone, which undergoes cyclization with isomerase, and a flavanone is produced—a starting compound for synthesis of the remaining groups of flavonoids [3,4].

All naturally found flavonoids have three hydroxyl groups: two found in ring A at position 5 and 7, and one in ring B at position 3. Diverse positions of substituents in a flavonoid molecule give it different chemical and physical properties, which translates into an individual metabolism of a given compound and its biological activity. Flavonoids, except for isoflavones, have their aromatic ring attached to pyrone C2, whereas in isoflavones it is attached to C3 [3,4,5,6,7].

**Figure 2:**
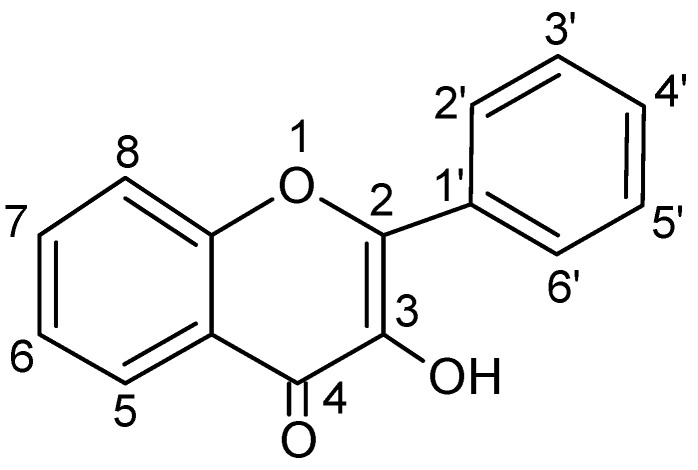
Basic skeleton or structure of flavonols.

**Figure 3:**
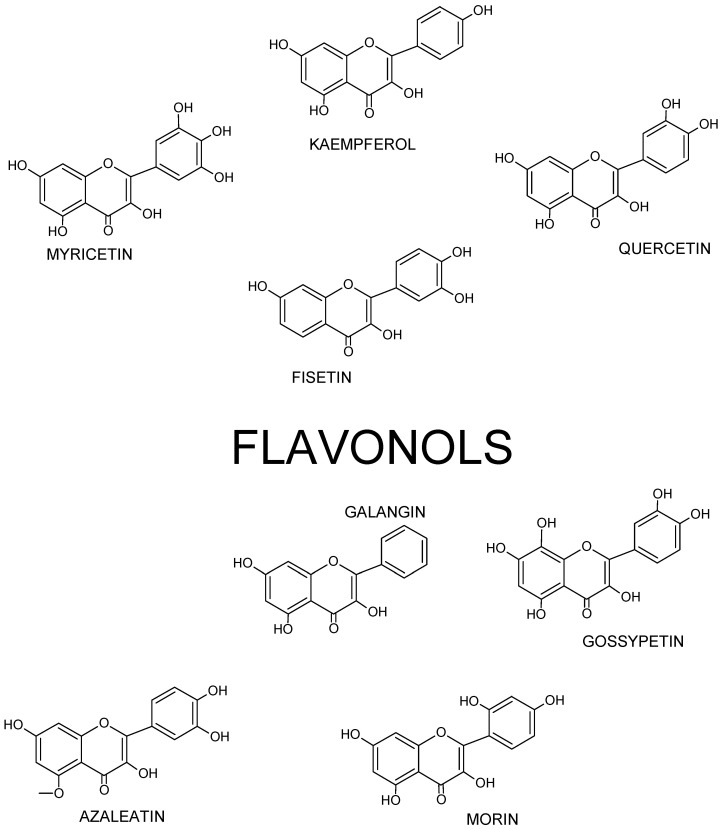
Structures of selected flavonols.

Chemically, flavonols differ from many other flavonoids, because they have a double bond between position 2 and position 3 as well as oxygen (a ketone group) at position 4 of ring C similarly to flavones, from which they yet differ due to the presence of hydroxyl group at position 3 (Figure 2 and Figure 3).

Thus, flavonol’s skeleton is 3-hydroxyflavone. 3′ hydroxyl group may attach sugar, i.e., it might be glycosylated. It was shown that three structural groups are important for biological activity, including anti-oxidative and radical-scavenging activity of flavonoids: (1) o-dihydroxyl group in ring B; (2) double bond at 2,3 attached to 4-oxy group in ring C; and (3) hydroxyl groups at positions C3 and C5. Moreover, a 3′,4′-o-dihydroxyl group is an indicator of a considerable antioxidant activity of flavonoids, especially those that do not belong to the flavonol subclass. Ring B catechol group is critical for high antioxidant activity. A higher antioxidant activity of flavonoids with o-dihydroxyl group in ring B is attributed to their greater radical stability via an increased delocalization of electrons and intramolecular hydrogen bonds between 3′- and 4′-hydroxyl groups. Additionally, 5′-hydroxyl group in ring B, as seen in myricetin and azaleatin, decreases the antioxidant activity, which might be caused by a prooxidant activity introduced by a pyrogallol group. [11,12,13].

## 3. Anticancer Potential of Flavonols

### 3.1. Head and Neck Cancer

Epidemiological data indicate that neoplasms regarding head and neck organs are more common in men (about 66–95% of cases), and the morbidity rate with regard to sex differs depending on anatomical site and changes with an increasing percentage of smoking women. Currently, the rate is 3:1 for oral cavity and oropharyngeal cancers. Prevalence of head and neck cancers increases with age, especially after the age of 45, and the peak for incidence is observed between the age of 50 and 70; however, these cancers also develop in younger individuals.

Their prevalence increases in poorly developed countries and in countries, in which resources are limited. These tumors are regarded as an important issue for public health in developed and developing countries. They are an important reason behind death of people due to their ability to disrupt basic life functions, such as breathing, speaking, hearing, seeing, smelling, or swallowing.

Neoplasms of this type are considered malignant; they develop on the mucous membrane of the air and digestive tracts and, thus, may be called cancers. They be appear in the following among other sites: the oral cavity, salivary glands, nasal cavity, paranasal sinuses, nasopharynx, hypopharynx and oropharynx, thyroid, larynx, ear, and lip. Due to high probability of metastases to neck lymph nodes, and in most cases a small risk of developing distal metastases (apart from the thyroid cancer), they are classified as locally malignant. Moreover, they are believed to be a group of highly heterogenic malignant tumors because their responsiveness to treatment and clinical course are conditioned to a large extent by their site.

Squamous cell carcinoma (SCC) is considered to be the most common histological type as it constitutes over 90% of cancers in those areas. In most cases, these are cancers found within the oral cavity, including the tongue and larynx (about 60–70%) [14,15,16,17,18]. Over 95% of cases are SCCs that are either well or moderately differentiated. Adenocarcinomas originating in small salivary glands are relatively rare.

In 2020, which is the last year with available data for morbidity, in worldwide there reported over 930,000 head and neck cancers, that is presented in Table 1.

### 3.2. Flavonols as Anticancer Agents

Dietary factors play a significant role in preventing neoplastic diseases. Fruit and vegetable containing flavonoids are described as antineoplastic agents. Consumption of onions and/or apples, two important sources of flavonols, is associated with more sporadic incidence of prostate, lung, stomach, and breast cancers. Furthermore, it appears that individuals who drink wine moderately show a lower risk of developing lung, endometrium, esophagus, stomach, and colon cancers. A connection between fruit and vegetable intake with cancer prophylaxis has been documented precisely. It has been suggested that considerable benefits for public health can be achieved by increasing consumption of this food.

Cancer development is a multi-stage process that involves initiation, promotion and progression. Flavonols present in the diet might affect and modulate a number of different biochemical processes and pathways participating in carcinogenesis. Further, they can act of biological response modifiers that support functioning of the immune system and protect live cells by being damaged by free radicals. Natural flavonols emerge as the main aim in searching for new antineoplastic therapies.

Antioxidant function of these compounds, which prevents various degenerative diseases associated with oxidative stress, including neoplastic diseases, is considered to be the main mechanism of their activity. This ability stems from reductive properties of flavonols and their metabolites. They also disrupt oxidation reaction thanks to phenol groups present in their structure that create stable phenoxyl radicals by receiving electrons. In addition, these compounds intrinsically have prooxidative properties, which they utilize to induce apoptosis and thereby inhibit cancer development [20].

Several mechanisms of the flavonol influence on the stages of initiation and promotion of carcinogenesis have been proposed. The main molecular mechanisms of flavonoid activity as follows:

- down-regulation of mutant p53 protein;
- stimulation of the apoptotic process;
- inhibition of the cell cycle;
- inhibition of heat shock proteins;
- estrogen receptor binding;
- inhibition of Ras protein expression.

Moreover, several other mechanisms preventing neoplasms that are affected by flavonols have been identified, including prevention of oxidation, detoxification of xenobiotics, and anti-inflammatory properties as well as their influence on the cellular signaling system [21].

Antineoplastic mechanisms of polyphenols include also their ability to modify activity of signal pathways engaged in proliferation of neoplastic cells. For instance, they include the mitogen-activated protein kinase (MAPK) pathway and phosphatidylinositol 3-kinase (PI3K) pathways. In order to inhibit the cell cycles, polyphenols disrupt the inhibition process of cyclic adenosine monophosphate (cAMP) or extracellular signal-regulated kinase 1 and 2 [20].

#### 3.2.1. Fisetin in Head and Neck Cancer

Fisetin (3,7,3′,4′-tetrahydroxyflavone) is a dietary flavonoid found in various fruit (strawberries, apples, mangos, persimmons, kiwis, and grapes), vegetables (tomatoes, onions, kale, and cucumbers), nuts, and wine [22]. However, it should be highlighted that this flavonol exists in nature in low concentration (up to hundreds of micrograms per 1 g of fresh biomass). There is a reason why this compound has become interesting. Namely, it has been recently observed that it is not only an effective antioxidant agent, but it also shows exceptional selectivity with regard to many biological processes considered as essential for biological homeostasis [23]. Studies showed strong anti-inflammatory, antioxidant, antineoplastic, anti-invasive, antiangiogenic, antidiabetic, and neuro- as well as cardio-protective activities in cell cultures and animal models [22,24,25].

Evaluation of cytotoxicity of examined compounds against cells in in vitro conditions is an important aspect of general toxicology studies, which are necessary to determine correctly the activity and safety of substances. Various cell tests are used for quantitative determination of cytotoxic activity of chemotherapeutic compounds. It is one of the first stages of examining biologically active substances.

Toxicology studies on fisetin showed that it generally decreases cell viability in a manner dependent on the dose and time in the studied cell lines. It was shown that it cytotoxically affects cell line Ca9-22 of gingival squamous cell carcinoma and cell line CAL-27 of the tongue carcinoma [26]. It is worth noting that this study demonstrated that Ca9-22 cells were relatively resistant to the same doses as compared with CAL-27 cells, and an approximate IC_50_ value was 50 and 200 µM, respectively, for CAL-27 and Ca9-22 cells after 48 h of incubation. The studies showed that fisetin also decreased total viability of tongue squamous cell carcinoma cell line HSC3 depending on the dose and cell exposure time, while IC_50_ value equaled 40 µM in case of the gum cancer cells [27]. Other researcher used the same cell line and showed that IC_50_ value was lower more than half and equaled about 20 µM (after 24 h) [28]. Fisetin also decreases viability of SCC-4 cells depending on the dose and time, with IC_50_ equaling to 52.8 µM after 48 h of treatment [29]. Studies conducted on human cell clines of the laryngeal cancer, including TU212, Hep-2, and M2e, showed that fisetin IC_50_ value equaled to about 10 µM. These cells appear to be the most sensitive to fisetin’s activity [30], which is presented in Table 2.

Understanding the mechanism lying at the bottom of apoptosis is important because it plays a key role in pathogenesis of numerous diseases, and its role has not been fully discovered. Regardless of the type of apoptotic course, ones of the main elements combining all these pathways are caspases, which depending on the stage of apoptosis in which they participate can be divided into initiator and effector caspases. Apoptosis is an orderly and organized cell process, which occurs in physiological and pathological conditions. It is also one of the most common topics for examination among cell biologists. An ability of a cell to conduct this process is significant for the carcinogenetic process. Apoptotic mechanism is complex and involves many paths. Defects may appear in any site within these pathways and lead to malignant transformation of the affected cells, tumor metastases, and resistance to antineoplastic drugs [31].

The beginning of the apoptotic process is characterized by cell and nucleus shrinkage as well as nuclear chromatin condensation into sharply differentiated masses, which are cut from nuclear membranes. Then, the cell nucleus gradually condenses and becomes fragmented (karyorrhexis). The cell separates itself from the surrounding tissue, its contours become undulated and extensions are created. Such changes in cell morphology were shown in CAL-27, Ca9-22 [26], and HSC-3 [27,28] cell lines treated with fisetin. In some cases these effects were time-dependent. Thus, it was shown that fisetin causes DNA damage in cells (fragmentation of the cell nucleus) [26,27]. Other studies showed that exposure of TU212 cells to fisetin causes necrotic morphological changes and decreases the percentage of live cells by means of cytotoxicity. However, more detailed studies showed that, after all, the key role in this process was played by apoptosis [30].

One of the most important regulators of the intrinsic apoptotic pathway is a family of Bcl-2 proteins. These proteins includes pro-apoptotic pore-formers (BAX, BAK, and BOK) and pro-apoptotic BH3-only proteins (BAD, BID, BIK, BIM, BMF, HRK, NOXA, PUMA, etc.) as well as anti-apoptotic factors, such as BCL-2, BCL-XL, BCL-W, MCL-1, and BFL-1/A1 [26,32].

**Figure 4:**
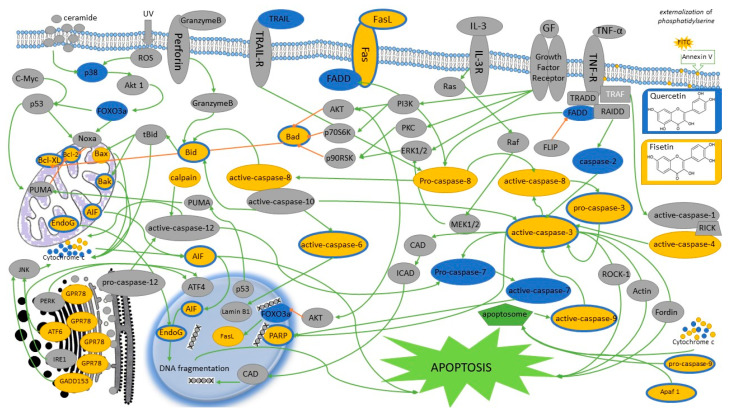
The figure shows the proposed signaling pathways of quercetin- and/or fisetin-induced apoptosis in head and neck cancer cells. Yellow indicates apoptotic signaling pathways in which fisetin is involved; the trails in which quercetin takes part are marked in blue or blue. Gray indicates proteins whose influence has not been tested or confirmed. Additionally, blocking signals are marked with amber arrows. AIF, apoptosis Inducing Factor; AKT, protein kinase B; Akt1, AKT serine/threonine kinase 1; Apaf-1, apoptotic protease activating factor 1; ATF4, activating transcription factor 4; ATF6, activating transcription factor 6; ATF-6α/β, activating transcription factor 6α/β; Bak, BCL-2-antagonist/killer; Bad, BCL2 associated agonist of cell death; Bax, BCL2 associated X; Bcl-xL, B-cell lymphoma-extra large; Bcl-2, B cel lymphoma 2; Bid, BH3 interacting-domain death agonist; CAD, caspase-activated DNase; c-Myc, proto-oncogene c-Myc; Cyto c, cytochrome c; ER, endoplasmic reticulum; Endo G, endonuclease G; ERK1/2, extracellular signal-regulated kinase ½; FADD, fas-associated protein with death domain; FLIP, FLICE-inhibitory protein; Fas, fas cell surface death receptor; FasL, Fas ligand; FOXO3a, forkhead box O3; GADD153, growth arrest- and DNA damage-inducible gene 153; GF, growth factor; GPR78, G protein-coupled receptor 78; GRP-78, glucose regulated protein 78; ICAD, inhibitor of CAD; IL-3R, human interleukin 3 receptor; IRE1, Inositol-requiring enzyme 1; JNK, c-Jun N-terminal kinase; MEK1/2, mitogen-activated extracellular signal-regulated kinase; Noxa, phorbol-12-myristate-13-acetate-induced protein 1; PARP, poly (ADP-ribose) polymerase; PERK, protein kinase R (PKR)-like endoplasmic reticulum kinase; PI3K, phosphoinositide 3-kinase; PKC, protein kinase C; PUMA, p53 upregulated modulator of apoptosis; p53, nuclear transcription factor; p70S6K, ribosomal protein S6 kinase beta-1; p90RSK, P90 ribosomal S6 kinase; Ras, binary switches, cycling between ON and OFF states during signal transduction; Raf, proto-oncogene serine/threonine-protein kinase; RAIDD, RIP-associated Ich-1/Ced-3-homologue protein with a death domain; RICK, receptor interacting serine/threonine kinase 2; ROCK-1, rho-associated, coiled-coil-containing protein kinase 1; ROS, reactive oxygen species; tBid, truncated BID; TNFα, tumor necrosis factor α; TNF-R, tumor necrosis factor receptor; TRADD, tumor necrosis factor receptor type 1-associated DEATH domain protein; TRAF, TNF receptor-associated factor 2; TRAIL, TNF-related apoptosis-inducing ligand; TRAIL-R, TNF-related apoptosis-inducing ligand receptor; UV, ultraviolet.

Fisetin increases the activity of caspase-3, -8, and -9 in cells of head and neck cancers. Moreover, fisetin considerably increases the expression of the following proteins: BID, BAD, BAK, BAX, AIF, ENDOG, cytochrome c, APAF1, cleaved form of caspase-9, cleaved form of caspase-3, caspase-6 and PARP, FAS, FAS ligand, and cleaved form of caspase-8, caspase-4, fragmented form of ATF-6β, ATF-6 α of calpain-1 and 2, caspase-4, GRP78, and GADD153; however, it decreases the expression of MCL1, BCL2, BCL-x, and XIAP (Figure 4). It might suggest that fisetin induced apoptotic death of HSC3 cell line via ergastoplasm stress and mitochondria-dependent pathways. Fisetin is also responsible for translocation of proteins associated with the apoptope in HSC3 cells, and an increased release of cytochrome c, AIF, and ENDOG from mitochondria into the cytoplasm [19,27]. Subsequent studies confirm that treatment with fisetin causes apoptosis; however, this time in OSCC cells with PAK4 overexpression. These conclusions were proposed by examining activation of PARP cleavage and caspase-3, which are characteristic features for apoptosis. Western blot analysis showed an increased PARP cut and an increased cleavage of caspase-3 fragments [33]. An increased PARP cleavage was also observed in case of HSC3 cells treated with fisetin [28]. Fisetin in high concentrations induces apoptosis and stimulates activation of cleaved caspase-3 and caspase-9, which leads to an increased PARP cut in Tu212 cells. Furthermore, fisetin increases expression of the BAX protein and weakens the level of Bcl-2 [30].

**Figure 5:**
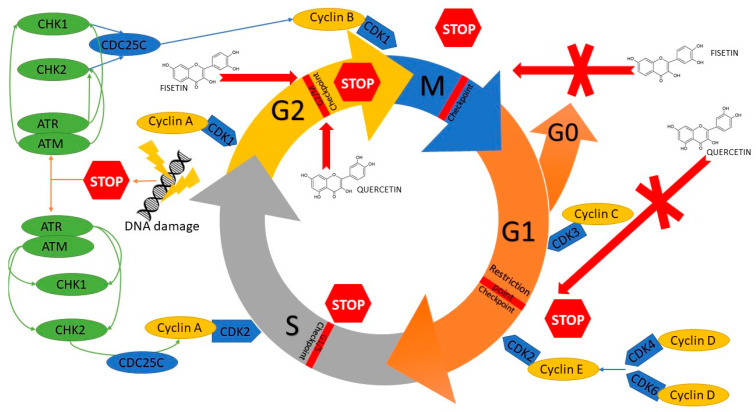
Mechanism of cell cycle inhibition of head and neck cancer cells by modulation of several regulatory proteins under the influence of quercetin and fisetin. CDC25C, M-phase inducer phosphatase 3; CDK1, Cyclin-dependent kinase 1; CDK2, Cyclin-dependent kinase 2; CDK3, Cyclin-dependent kinase 3; CDK4, Cyclin-dependent kinase 4; CDK6, Cyclin-dependent kinase 6; CHK1, Checkpoint kinase 1; CHK2, Checkpoint kinase 2; ATM, serine/threonine kinase; ATR, Serine/threonine-protein kinase.

Moreover, the studies showed that fisetin induces production of reactive oxygen species (ROS), increases Ca2+ release, and decreases the mitochondrial membrane potential (Ψm) in head and neck neoplastic cells. The results may suggest that ROS, Ca2+ and ΔΨm participate in fisetin-induced apoptosis of HSC3 [27] and SCC-4 [29] cells. Fisetin induces G2/M phase cell cycle arrest and induces the sub-G1 phase. Moreover, it was shown that fisetin inhibits expression of CDC25C proteins, cyclin A and B, CDK1, and CDK2; it also increases the expression of CHK1 and 2 proteins, which are connected to G2/M phase arrest [29]. Fisetin inhibits the cell cycle in cells with PAK4 overexpression probably by arresting cells in the G2/M phase and not letting them reach phase S, i.e., DNA synthesis (Figure 5) [33].

Receptor tyrosine kinase, c-Met, may undergo overexpression in many neoplasms and is of key importance for their biological and chemical functions. c-Met activation leads to an increased growth of cells, invasion, angiogenesis, and ability to create metastases. Hepatocyte growth factor (HGF), synthetized by fibroblasts, is one of the ligands for this receptor. HGF plays an important role in carcinogenesis. HGF blockade inhibits c-Met kinase’s signal transmission. Sustained c-Met activation may mediate resistance to c-Src inhibition. The differences between c-Met and c-Src signaling in sensitive and resistance cells result from distinct factors promoting or inhibiting interactions, rather than from intrinsic structural changes in c-Src or c-Met. The synergistic cytotoxic effects of c-Src and c-Met inhibition may be important for the treatment of head and neck cancers [2].

Research demonstrated that fisetin inhibits c-Met/Src receptor signaling and inhibits PAK4 kinase expression in both SCC9 and SCC4 cells. High levels of PAK4 protein promote proliferation and/or survival of cells. Furthermore, it was shown that fisetin considerably inhibits colony formation of PAK4-overexpressing OSCC cells, and considerably reduces the ability of SCC9-PAK4-Lv and Tca113-PAK4-Lv cells to migrate [33]. Furthermore, it was shown that initial treatment of TU212 cells with fisetin promotes inhibition of colony formation, which leads to a considerable reduction in the colony formation factor as compared with the group [30]. Then, it was demonstrated that the total quantity of Met, Src, p-Met, and p-Src proteins was considerably reduced by fisetin, which suggests that tyrosine phosphatases are engaged in inhibition of the Met/Src signaling pathway, which is mediated by fisetin. In addition, it was proven that ADAM9 protein might participate in several pathways associated with carcinogenesis, including ITGAV, UB, and TNF pathways. What is more, fisetin inhibits several proteins, including ADAM9, MTOR, AR, and CDK6, and it down-regulates ADAM9 expression in OSCC cells, which inhibits formation of tumors and invasiveness of several types of neoplastic cells [33,34].

Apoptosis induced by fisetin administration occurs by caspase-3 overexpression and regulation of PI3K/Akt/NF-κB signaling pathway. In addition, fisetin inhibits proliferation of TU212 cells, which is associated with inactivation of ERK1/2 kinase. Fisetin inhibits activation of mTOR regulated by PI3K/AKT, which leads to suppression of transcription and inhibition of TU212 cell proliferation. Data shows that fisetin play a potential role in controlling human laryngeal cancer by inhibition of neoplastic cell proliferation, and induction of apoptosis and autophagy regulated by ERK1/2 and AKT/NF-κB/mTOR signaling pathways, which may constitute a therapeutic strategy for the larynx and inhibition of cancer in the future [30].

It was also shown that fisetin affects over 140 genes increasing the level of 61 and decreasing the level of 81 genes. A considerable increase in SESN2 protein expression in HSC3 cells treated with fisetin was observed, whereas CHAC1 protein did not change. It was shown that SESN2 mediates apoptosis induced by fisetin in HSC3 cells. Additionally, an influence of fisetin on mTOR kinase and Mcl-1 protein was determined, and it was shown that fisetin decreases the levels of p-mTOR and Mcl-1 protein expression. Results suggest that the apoptosis induced by fisetin might be associated with SESN2/mTOR/Mcl-1 signaling axis [28].

#### 3.2.2. Kaempferol in Head and Neck Cancer

Kaempferol (3,5,7-trihydroxy-2-(4-hydroxyphenyl)chromen-4-one) is a type of flavonoid belonging to the flavonol group. It is soluble in hot ethanol as well as alkaline ether, and poorly soluble in water. Kaempferol has hydrophobic properties thanks to its diphenylpropane structure. Kaempferol is found in various plant parts, such as seeds, leaves, fruits, flowers, and even in vegetables [35,36]. Kaempferol can be isolated from tea, and also many other popular vegetables and fruit, including beans, broccoli, cabbage, gooseberries, grapes, kale, strawberries, tomatoes, citrus fruits, Brussels sprouts, apples, and grapefruits. It was also identified in various medicinal plants, including *Equisetum* spp., *Sophora japonica*, *Ginkgo biloba,* and *Euphorbia pekinensis* (Rupr.). The most known property of kaempferol is its anti-inflammatory activity [35]. Moreover, kaempferol and its glycosylated derivatives have the following effects: cardioprotective, neuroprotective, anti-inflammatory, antidiabetic, antioxidant, and antibacterial [36]. One of the most important properties of kaempferol is its antineoplastic activity towards cancers affecting the following: esophagus [37], pharynx [38], breast [39], liver [40], ovary [41], stomach [42], lung [43], pancreas [44], bladder [45]; and osteosarcoma [46] as well as leukemia [47]. However, specific mechanisms of kaempferol’s activity against many types of cancers remain unknown.

Studies showed that kaempferol dose-dependently decreases cell viability in FaDu line pharyngeal cancer. In 0.1 µM and 1 µM concentrations, kaempferol is more effective than apigenin in decreasing cell viability. IC_50_ value of kaempferol after 48 h is about 46 µM. Studies involving PCI-13 and PCI-15B lines showed that the activity of apigenin and kaempferol is more distinct after 48 h of incubation. Treating PCI-15B cells with kaempferol causes a two-phase response, in which lower concentrations (i.e., 10 µM and 25 µM) resulted in an increase of cell numbers, whereas a decrease in cell viability was observed with higher concentrations (i.e., 100 µM and 200 µM) [38]. It was demonstrated that IC_50_ value of kaempferol in relation to SCC-1483 cell line was approximately 40 µM after 24 h of incubation [48]. Studies of Lin CW [49] appear to be interesting due to the fact that cytotoxic activity of kaempferol was not demonstrated in 0–100 µM concentrations in relation to tongue cancer cell line SCC-4.

Kaempferol induces apoptosis in SCC-1483 cells treated with various concentrations of kaempferol. Apoptosis was induced in cells and its effect was dose-dependent [48]. In order to confirm an effect of kaempferol on the induction of apoptosis, other cells lines, including SCC-25 and SCC-QLL1, were examined as well. Apoptosis was induced in all neoplastic cell lines treated with kaempferol. Moreover, caspase-3 activity test showed that the induction of apoptosis by kaempferol depends on caspase-3 [48].

Furthermore, it was demonstrated that kaempferol essentially inhibits migration and invasion of SCC4 cells in a dose-dependent way, and that it is responsible for the inhibition of MMP-2 enzyme activity with up to 53% at the highest concentration of kaempferol (i.e., 100 µM). TIMP-2 protein level was decreased under the influence of kaempferol. These results were confirmed by the assessment of mRNA expression and it was demonstrated that kaempferol considerably inhibits mRNA MMP-2 expression that is concentration-dependent. Further, the activity of MMP-2 promoter was decreased in a dose-dependent way, which indicates that kaempferol inhibits MMP-2 expression at the transcription level. Additionally, it was demonstrated that activity of the AP-1 bond with MMP-2 promoter decreases under the influence of kaempferol in a concentration-dependent way, and the ability of AP-1 bond on the MMP-2 gene promoter is decreased. Treating SCC4 cells with kaempferol also decreases c-Jun protein translocation to the nucleus; however, it does not affect the level of c-Fos protein. Kaempferol inhibits SCC4 cell migration by reducing MMP-2 expression and suppresses ERK phosphorylation; however, it does not affect the phosphorylation of JNK1/1 and p38 pathways. Suppression of MMP-2 expression by kaempferol occurs mainly by inhibiting the ERK1/2 signaling pathway [50].

#### 3.2.3. Quercetin in Head and Neck Cancer

Studies conducted on cell lines of neoplasms affecting the area of head and neck showed that these cells are sensitive to quercetin, and the sensitivity depends on the cell line, exposure time and quercetin concentration. It was demonstrated that is cytotoxically affects the following cell lines of the tongue cancer: SAS [51,52,53,54], OSC20 [52], SCC25 [48,55,56], SCC-9 [57], HSC-3 [58,59], Tca8113 [49,54], SCC-15 [49], TW206 [58]; throat cancer: FaDu line [59]; oral cavity cancer: HN22 [52] and TW206 [58] lines.

IC_50_ value of quercetin in relation to tongue cancer cell lines was variable and equaled from 20 μM in case of HSC-3 cell line up to 160 μM in relation to SAS and OSC20 cell lines (Table 3).

Like during the studies on fisetin, an influence of quercetin on the morphology of the examined cells was evaluated. Observations conducted with the use of a light microscope showed that quercetin leads to morphological changes in cells, including cell shrinkage [51], shape changes, cellular edema, and cell plasma damage might indicate necrosis and/or apoptosis [57]. Then, condensation and fragmentation of nuclear chromatin was observed in HSC-3 and TW206 neoplastic cells lines what may indicate an induction of programmed cell death—apoptosis [58]. Moreover, it was demonstrated that initial several-hour-long incubation of cells with quercetin considerably increases the percentage of apoptotic cells treated with cisplatin. This mechanism can rest on increasing cell sensitivity to cisplatin after using quercetin [49]. Quercetin induces cell apoptosis, which is shown by an increase in percentage of early apoptotic cells in a time-dependent way [51,54]. Quercetin is also responsible for an increase in reactive oxygen species and Ca2+ production, a decrease in the level of the mitochondrial membrane potential (ΔΨm), and a change in expression of proteins associated with apoptosis. Quercetin increases expression of caspase-2, Bak, Bid and Bad, cyt c, Apaf-1, Endo G, AIF and PARP, active caspase-9, caspase-3, caspase-6 and caspase-7, TRAIL, Fas-L, Fas, FADD and active caspase-8, ATF-6α, ATF-6β, XBP- 1, IRE-1α, caspase-4 and GRP-78; however, it inhibits expression of Bcl-2 and Bcl-x, as well as pro-caspase-3. Furthermore, quercetin increases the expression level of cytochrome c, apoptosis inducing factor and endonuclease G, which are directly associated with apoptotic pathways [51]. Quercetin significantly decreases the level of anti-apoptotic Bcl-2 oncoprotein, while increasing the level of proapoptotic Bax protein. Proteins from the Bcl-2 family and Bax proteins mediate the mitochondrial pathway of apoptosis. The ration of Bcl-2 to Bax proteins affects the susceptibility of cells to induction of apoptotic cell death. Quercetin also increases the expression of the active caspase-3 (cleaved caspase-3) and poly (ADP-ribose) polymerase (cleaved PARP) in SCC-25 and SCC-9 cell lines of the tongue cancer (Figure 5) [55,57].

Inducing an inhibition of a cell cycle and apoptosis are well-known methods for treating some types of cancer. It was demonstrated that quercetin causes an increase in the percentage of cells in the G2/M phase of the cell cycle and a decrease in the percentage of cells in the G0/G1 phase [52,58]. After a treatment involving various doses of quercetin (25–75 μM), the percentage of cells in the G1 phase increases with a simultaneous decrease in the number of cells in the S phase and a gradual increase in the percentage of cells in the G1 phase with simultaneous increases in the cells in the sub-G phase, which indicates induction of apoptosis (Figure 5) [55]. However, some scientific reports show that quercetin increases the population of cells in the S phase with a respective decrease in the number of cells in the G1 phase in a dose-dependent way and increases the non-significant population in the G2-M phase. These results might be caused by an inhibiting influence of quercetin on DNA synthesis [57]. Quercetin is also responsible for a decrease in the number of G2/M phase cyclins, i.e., cyclin A and cyclin B1; however, it does not affect cyclin D1, i.e., G1/S phase cyclin. What is more, it induces p21 protein expression, but does not affect the expression of p15 protein—an inhibitor of G1/S [58].

Quercetin also exerts an inhibiting effect on cell migration [52,53]. Dose-dependently, it increases expression of epithelial markers, such as E-cadherin and claudin-1, while simultaneously decreases the expression of mesenchymal markers, such as fibronectin, vimentin, and alpha-smooth muscle actin (α-SMA). It reduces specific expression of mesenchymal markers in OSCC cells. EMT-activating transcription factors, such as Twist, Slug, and Snail 1 are significantly reduced after the use of quercetin [52].

It is well known that many stages and connected factors are involved in migration and invasion of neoplastic cells. Studies demonstrated that quercetin and other polyphenols might inhibit matrix metalloproteinase (MMP) activity, especially MMP-2 and MMP-9. These two key regulators associated with invasion and metastases might be activated by E2F transcription factor, which plays an essential role in cell proliferation by regulating expression of genes involved in G1/S transition and DNA synthesis [55]. It was demonstrated that quercetin inhibits not only migration, but also invasion of in vitro cells [53]. Moreover, quercetin inhibits MMP-9 and MMP-2 expression and reduces levels of the following proteins: MMP-2, -7, -9 [49,53] and -10 as well as VEGF, NF-κB p65, iNOS, COX-2, and uPA, PI3K, IKB-α, IKB-α/β, p-IKKα/β, FAK, SOS1, GRB2, MEKK3 and MEKK7, ERK1/2, p-ERK1/2, JNK1/2, p38, p-p38, c-JUN, and pc-JUN; however, it does not affect RhoA, PKC, and RAS. It was noted that ERK signaling pathway up-regulated MMP expression. Moreover, quercetin inhibits ERK1/2 phosphorylation in cells, which indicated that quercetin might inhibit the ERK signaling pathway. It was also demonstrated that quercetin affects the level of RhoA and NF-κB proteins in SAS cells, and stimulates the expression of RhoA, ROCK1, and NF-κB in SAS cells [53].

Improper activation of EGFR is the main reason behind malignancy of the oral cavity cancer. In HSC-3 cells with EGFR overexpression, the level of p-Y1086-EGFR protein and its p-Akt effector decrease due to quercetin both with time and dose, while p-Y1045-EGFR levels increase. It is worth noting that phosphorylation of the tyrosine 1045 is responsible for ubiquitination mediated by c-Cbl and degradation of EGFR protein. Quercetin is an important modulator of EGFR/Akt pathway. FOXO transcription factors play a significant role in regulating cell growth and apoptosis. Activity of FOXO factors might be regulated by excluding the nucleus that is medicated by Akt and subsequent degradation. Quercetin causes an increase in the level of FOXO1 protein both in a dose- and time-dependent way; however, it does not affect changes in expression of FOXO3a. Quercetin reduces phosphorylation of cytoplasmic FOXO1 in serine 256 and induces translocation of FOXO1 from the cytoplasm into the nucleus [58].

#### 3.2.4. Future of Flavonoid Research

At present, despite numerous basic studies using in vitro and in vivo models, there are insufficient data to confirm the health impact of most flavonoid subclasses. Future studies of the bioactivity of flavonoids require a more complete understanding of their bioavailability, metabolism, and elimination. The future of flavonoid research will undoubtedly depend on resolving the standardization problems of flavonoid research, and although the field shows continuous progress, it may slow down in the coming years if these challenges are not met [60]. Few studies suggest that the consumption of flavonoids is associated with a lower risk of head and neck cancers. The evidences suggest that dietary flavonoids could potentially be used as a risk reduction strategy in people at high risk for head and neck cancer to reduce the incidence of these very fatal cancers. However, further laboratory, clinical, and epidemiological studies are warranted before a final conclusion about the chemopreventive potential can be drawn [61].

Determining the biological activity of phytotherapeutic agents (including flavonols) depends on a full understanding of the processes: consumption, absorption, metabolism, and excretion; however, this has only been done for a handful of molecules so far. The ability to scavenge plasma free radicals after flavonoid supplementation indicates that a significant amount of previously unknown metabolites must be present in the blood, implying a much greater absorption and bioavailability than previously perceived. These unknown metabolites are still a subject of much debate and most likely contribute to the bioactivity of the flavonoids. Scientists should focus less on the parent compounds at the time of ingestion, and more on the biological activity of metabolites present in our tissues. They show the strongest biological properties in our body [60]. In order to define more important chemical structures of promising compounds or compound classes, it is necessary to conduct a systemic analysis of structure-activity dependencies. Such research may help design, modify, and develop new flavonols as potential antiproliferative measures used against neoplastic cells. Many studies have emphasized that molecular docking research is needed to identify potential flavonols’ molecules for their use in the treatment of various ailments in the human health system. It is now recommended to eat fruits, vegetables, and beverages that contain flavonols, although it is too early to make recommendations for a daily intake of flavonols.

## 4. Conclusions

Natural products arise considerable interest because they are easy to obtain and cause few side effects. A lot of convincing evidence indicated that flavonols play a promising role in preventing and treating many chronic diseases, such as cancers. It has been shown that they modulate various cellular and molecular signal transduction pathways; however, these mechanisms are complex and have not been fully explained yet. Further, a number of flavonols with different structures were examined. Still, our current understanding of dependencies between their chemical structure and antineoplastic properties is very limited, especially with regard to cancers in the area of head and neck. The interaction of flavonols with receptor molecules in the treatment of head and neck tumors is an important area of future research.

The biggest limitation in flavonoid research is the “superficial” description of bioactivity based on studies using flavonoids in non-physiologically relevant forms or concentrations. The future of flavonoid research therefore requires a multidisciplinary approach, including epidemiology, human intervention, and cell/molecular research.

Although little data on the concentration of flavonoids in human tissues is available, flavonoids have been shown to play an important role in antioxidant defense in both cells and tissues. Taking into account the highest concentration of flavonoids in the plasma in humans, which usually after 1–2 h from eating foods rich in flavonols (present in apples, onions, and buckwheat tea) is maximum 7 µM [62], it can be concluded that the commonly used dietary flavonoids are not able to show antitumor activity, but their consumption may prevent DNA damage leading to the development of neoplastic disease. However, keep in mind that their levels depend on the type of flavonoid, since anthocyanins and catechins have a half-life that is 5 to 10 times shorter than that of the flavonoids.

In conclusion, there is still controversy in research regarding the possible protective effect of flavonoids on cancer. Certain flavonoid subclasses appear to suggest a reduction in the risk of developing certain types of cancer. Given the importance of flavonoids in cancer chemoprevention as potential adjuvants and/or chemosensitizers, it is likely that long-term efforts will continue to investigate the effects and mechanisms of flavonoids in conjunction with standard chemotherapeutic agents. In particular, more clinical studies are needed to confirm the usefulness of flavonoids in patients.

## Figures and Tables

**Table 1:** Estimated number of incident cases and mortality factor in Europe (EURO) and Pan American (PAHO) caused by different anatomical types of tumors in the head and neck region, acc. to WHO [1,19].

	Incidence						Mortalilty					
	Worldwide		WHO Europe (EURO)		WHO Americas (PAHO)		Worldwide		WHO Europe (EURO)		WHO Americas (PAHO)	
	ENIC	ECI	ENIC	ECI	ENIC	ECI	END	ECM	END	ECM	END	ECM
Lip, oral cavity	377,713	4.8	69,856	7.5	45,357	4.4	177,757	2.3	26,530	2.8	12,533	1.2
Larynx	184,615	2.4	45,790	4.9	29,685	2.9	99,840	1.3	22,224	2.4	14,434	1.4
Nasopharynx	133,354	1.7	6820	0.73	4222	0.41	80,008	1	3537	0.38	2247	0.22
Orophargynx	98,412	1.3	30,061	3.2	22,910	2.2	48,143	0.62	13,664	1.5	8576	0.84
Hypopharynx	84,252	1.1	19,730	2.1	4892	0.48	38,599	0.5	9721	1	1740	0.17
Salivary glands	53,593	0.69	11,027	1.2	9791	0.96	22,778	0.29	4581	0.49	2399	0.23

Estimated crude incidence rate-ECI; Estimated crude mortality rate-ECM; Estimated number of incident cases-ENIC; Estimated number of deaths-END.

**Table 2:** Effects of fisetin in head and neck cancers.

Cancer Types	Cancer Cell Line	Effects	~IC_50_ Value	Protein/Molecule	Reference
Laryngeal	Hep-2	cancer growth ↓ proliferation ↓ apoptosis ↑ invasion ↓ metastasis ↓	10 µM (24 h)	Cleaved-Caspase-3 ↑ Cleaved-Caspase-9 ↑ Cleavage of PARP ↑ Bax ↑ Bcl-2 ↓ LC3II ↑ Raf ↓ Ras ↓ ERK1/2 ↓ Fos ↓ PI3K/AKT ↓ cIAP ↓ p-NF-κB ↓ TSC1 ↑ mTOR ↓	[30]
	TU212		15 µM (24 h)		
	M2e		10 µM (24 h)		
Laryngeal	UM-SCC-23	proliferation ↓ apoptosis ↑ RTK pathway ↓ Met/Src signaling pathways ↓		Met ↓ Src ↓ p-Met ↓ p-Src ↓ ADAM9 ↓ MTOR ↓ AR ↓ CDK6 ↓	[34]
Tongue	Tca-8113				
Tongue	SCC9 SCC4	proliferation ↓ Colony Formation ↓ Cell Cycle G2/M ↓ Migration ↓		PAK4 Expression ↓ cleavage of PARP ↑ cleavage of caspase 3 ↑	[33]
Tongue	CAL27	Apoptosis ↑ Ψm ↓ ratio of AVOs ↑	>120 µM (24 h) 50 µM (48 h) 40 µM (72 h)	Bcl-2 ↑ Bcl-XL ↑ Bax↓ Bak ↓ caspase-3 ↑ PARP ↑ p62 ↓ Bekliny-1 ↑ ATG5 ↑	[26]
Gingival	Ca9-22		200 µM (24 h) 180 µM (48 h) 140 µM (72 h)		
Gingival	HSC-3	Cytotoxic effect ↑ chromatin condensation ↑ DNA damage ↑ ΔΨm ↓ ROS ↑	20 µM (24 h) 50 µM (48 h)	caspase-3 ↑ caspase-8 ↑ caspase-9 ↑ BID ↑ BAD ↑ BAK ↑ BAX ↑ AIF ↑ ENDO G ↑ cytochrome c ↑ APAF1 ↑ cleaved caspase-9 ↑ cleaved caspase-3 ↑ caspase-6 ↑ PARP ↑ FAS ↑ FAS-ligand ↑ cleaved caspase-8 ↑ cleaved ATF-6β ↑ calpain 1 ↑ caspase-4 ↑ GRP78 ↑ MCL1 ↓ BCL2 ↓ BCL-x ↓	[27]
Tongue	SCC-4	G2/M arrest ↑ Sub-G1 ↑ chromatin condensation ↑ DNA fragmentation ↑ ΔΨm ↓ ROS ↑ Ca 2+ ↑	100 µM (24 h) 53 µM (48 h)	caspase-3 ↑ caspase-8 ↑ caspase-9 ↑ Calpain 1 and 2 ↑ Bax ↑ AIF ↑ Cytochrome c ↑ Bcl-2 ↓ MCL-1 ↓ XIAP ↓ GRP78 ↑ GADD153 ↑ caspase-4 ↑ ATF-6α ↑ ATF-6β ↑	[29]
Tongue	HSC3	Viability ↓ Apoptosis ↑ chromatin condensation ↑	15 µM (24 h)	PARP ↑ SESN2 ↑ CHAC 1 ↔ p-mTOR ↓ Mcl-1 ↓	[28]
Gingival	Ca9.22				

**Table 3:** Effects of quercetin in head and neck cancers.

Cancer Types	Cancer Cell Line	Effects	~IC_50_ Value	Protein/Molecule	Reference
Tongue	SAS	Cytotoxicity ↑ Apoptosis ↑ ROS ↑ ΔΨm ↓	40 µM (24 h)	activities of caspase-3 ↑ activities of caspase-9 ↑ activities of caspase-8 ↑ caspase-2 ↑ Bak ↑ Bid ↑ Bad ↑ Cyto c ↑ Apaf-1 ↑ Endo G ↑ AIF ↑ PARP ↑ active form of caspase-9 ↑ active form of caspase-8 ↑ active form of caspase-3 ↑ active form of caspase-6 ↑ active form of caspase-7 ↑ TRAIL ↑ Fas-L ↑ Fas ↑ FADD ↑ ATF-6α ↑ ATF-6β ↑ XBP-1 ↑ IRE-1α ↑ caspase-4 ↑ GRP-78 ↑ Bcl-2 ↓ Bcl-x ↓ pro-caspase-3 ↓ cytochromu c ↑ AIF ↑ Endo G ↑	[51]
Tongue	OSC20	Viability ↓ Cell Cycle G2/M Migration ↓	160 µM (24 h)	E-cadherin ↑ claudin-1 ↑ fibronectin ↓ vimentin ↓ 0-SMA ↓ MMP-2 ↓ MMP-9 ↓ Twist ↓ Slug ↓	[52]
Tongue	SAS		160 µM (24 h)		
Oral cavity	HN22		>200 µM (24 h)		
Tongue	SAS	Viability ↓ Cytotoxicity ↑ Migration ↓	75 µM (24 h)	MMP-2 ↓ MMP-9 ↓ MMP-7 ↓ MMP-10 ↓ VEGF ↑ NF-κB ↑ iNOS ↑ COX-2 ↑ uPA ↑ PI3K ↑ IKBα ↑ p-IKKα/β ↑ FAK ↑ SOS1 ↑ GRB2 ↑ MEKK3 ↑ MEKK7 ↑ ERK1/2 ↑ p-ERK1/2 ↑ JNK1/2 ↑ p38 ↑ p-p38 ↑ c-JUN ↑ pc-JUN ↑ RhoA ↔ PKC ↔ RAS ↔	[53]
Tongue	Tca8113	Viability ↓ Apoptosis ↑	49 µM (24 h)	miR-22 ↓ WNT1 ↓ β-kateniny ↓	[54]
	SAS		44 µM (24 h)		
Tongue	SCC-25	Viability ↓ Phase G1 cell cycle ↑ Apoptosis ↑ Migration ↓ Invasion ↓	50 µM (24 h)	cyclin D1 ↓ inp21Cip1 ↑ cyclin D1 ↑ Cip1/P21 ↑ Kip1/P27 ↑ Bcl-2 ↓ Bax ↑ cleaved caspase 3 ↑ cleaved PARP ↑	[55]
Tongue	SCC-9	cell growth ↓ cellular DNA synthesis ↓ apoptosis ↑ necrosis ↑		Caspase-3↑ Thymidylate synthase ↓	[57]
Tongue	HSC-3	cell growth ↓ Cell Cycle G2/M Chromatin condensation ↑	20 µM (24 h)	cyclin A ↓ cyclin B1 ↓ cyclin D1 ↔ p21 ↑ p15 ↔ FasL ↑ cleaved caspase 3 ↑ Bax ↔ p-Y1086-EGFR ↓ p-Akt ↓ p-Y1045-EGFR ↑ Akt ↓ EGFR ↓ FOXO1 ↑ FOXO3a ↔	[58]
	TW206		45 µM (24 h)		
Tongue	HSC-3	Migration ↓ Invasion ↓		MMP-2 ↓ MMP-9 ↓	[59]
Pharynx	FaDu				
Tongue	Tca-8113	Apoptosis ↑ colony formation ↓		Cleavage PARP ↑ caspase-3 ↑ caspase-9 ↑ caspase-8 ↑ xIAP ↓	[49]
	SCC-15				
